# How Does COVID-19 Pandemic Impact on Incidence of *Clostridioides difficile* Infection and Exacerbation of Its Gastrointestinal Symptoms?

**DOI:** 10.3389/fmed.2021.775063

**Published:** 2021-12-13

**Authors:** Masoumeh Azimirad, Maryam Noori, Hamideh Raeisi, Abbas Yadegar, Shabnam Shahrokh, Hamid Asadzadeh Aghdaei, Enrico Bentivegna, Paolo Martelletti, Nicola Petrosillo, Mohammad Reza Zali

**Affiliations:** ^1^Foodborne and Waterborne Diseases Research Center, Research Institute for Gastroenterology and Liver Diseases, Shahid Beheshti University of Medical Sciences, Tehran, Iran; ^2^Gastroenterology and Liver Diseases Research Center, Research Institute for Gastroenterology and Liver Diseases, Shahid Beheshti University of Medical Sciences, Tehran, Iran; ^3^Basic and Molecular Epidemiology of Gastrointestinal Disorders Research Center, Research Institute for Gastroenterology and Liver Diseases, Shahid Beheshti University of Medical Sciences, Tehran, Iran; ^4^Internal Medicine and Emergency Medicine, St'Andrea Hospital, Sapienza University, Rome, Italy; ^5^Department of Clinical and Molecular Medicine, Sapienza University, Rome, Italy; ^6^Infectious Diseases Service, University Hospital Campus Bio-Medico, Rome, Italy

**Keywords:** coronavirus disease 2019, *Clostridioides difficile* infection, antibiotic therapy, NSAIDs, gut microbiota, elderly, COVID-19

## Abstract

Coronavirus disease 2019 (COVID-19) has rapidly spread all over the world with a very high rate of mortality. Different symptoms developed by COVID-19 infection and its impacts on various organs of the human body have highlighted the importance of both coinfections and superinfections with other pathogens. The gastrointestinal (GI) tract is vulnerable to infection with COVID-19 and can be exploited as an alternative transmission route and target for virus entry and pathogenesis. The GI manifestations of COVID-19 disease are associated with severe disease outcomes and death in all age groups, in particular, elderly patients. Empiric antibiotic treatments for microbial infections in hospitalized patients with COVID-19 in addition to experimental antiviral and immunomodulatory drugs may increase the risk of antibiotic-associated diarrhea (AAD) and *Clostridioides difficile* infection (CDI). Alterations of gut microbiota are associated with depletion of beneficial commensals and enrichment of opportunistic pathogens such as *C. difficile*. Hence, the main purpose of this review is to explain the likely risk factors contributing to higher incidence of CDI in patients with COVID-19. In addition to lung involvement, common symptoms observed in COVID-19 and CDI such as diarrhea, highlight the significance of bacterial infections in COVID-19 patients. In particular, hospitalized elderly patients who are receiving antibiotics might be more prone to CDI. Indeed, widespread use of broad-spectrum antibiotics such as clindamycin, cephalosporins, penicillin, and fluoroquinolones can affect the composition and function of the gut microbiota of patients with COVID-19, leading to reduced colonization resistance capacity against opportunistic pathogens such as *C. difficile*, and subsequently develop CDI. Moreover, patients with CDI possibly may have facilitated the persistence of SARS-CoV-2 viral particles in their feces for approximately one month, even though the nasopharyngeal test turned negative. This coinfection may increase the potential transmissibility of both SARS-CoV-2 and *C. difficile* by fecal materials. Also, CDI can complicate the outcome of COVID-19 patients, especially in the presence of comorbidities or for those patients with prior exposure to the healthcare setting. Finally, physicians should remain vigilant for possible SARS-CoV-2 and CDI coinfection during the ongoing COVID-19 pandemic and the excessive use of antimicrobials and biocides.

## Introduction

Coronavirus disease 2019 (COVID-19) is a respiratory infection caused by the newly identified beta-coronavirus known as the severe acute respiratory syndrome coronavirus 2 (SARS-CoV-2) (1, 2). Although COVID-19 outbreak first occurred in Wuhan, a city in Hubei province of China on December 2019, it rapidly spread via human-human contact to the rest of the world (3, 4). When the virus infected people of 114 countries, COVID-19 was called a “pandemic” by the World Health Organization (WHO) (5, 6). On 8 November 2021, Center for Systems Science and Engineering (CSSE) at John Hopkins University confirmed around 250,799,409 cases so far throughout the world including 5,063,295 global deaths (https://coronavirus.jhu.edu/). COVID-19 patients may present with various clinical manifestations from an asymptomatic infection to a mild condition or acute respiratory distress syndrome (ARDS) and dysfunction, in different organs mainly the liver, bowel and kidneys (7, 8). Within most cases, fever, sore throat, cough, fatigue, dyspnea, sputum production, myalgia, fatigue, and headache occur. While such patients most commonly present with severe respiratory disease, it has been reported that about 19% of COVID-19 patients display gastrointestinal (GI) symptoms (1) including 2–10% diarrhea, 1–3.6% loss of appetite, nausea, vomiting, and abdominal pain that are increasingly being recognized as important symptoms of COVID-19 (9, 10). The virus has been detected in different parts of the patients' GI tract as well as the feces, suggesting the importance of the gut tropism in the pathogenesis and dissemination of this horrible pandemic (11). The overuse of empirical antimicrobial agents in COVID-19 patients increase the risk of microbiota alterations, antibiotic-associated diarrhea and *Clostridioides difficile* infection (CDI). Therefore, involvement of the GI tract in COVID-19 warrants attention of clinicians and researchers. This article also gives an update of the recent evidences on the gut microbiota disruption during COVID-19 and on the occurrence of CDI.

## SARS-CoV-2 Structure

**Figure 1:**
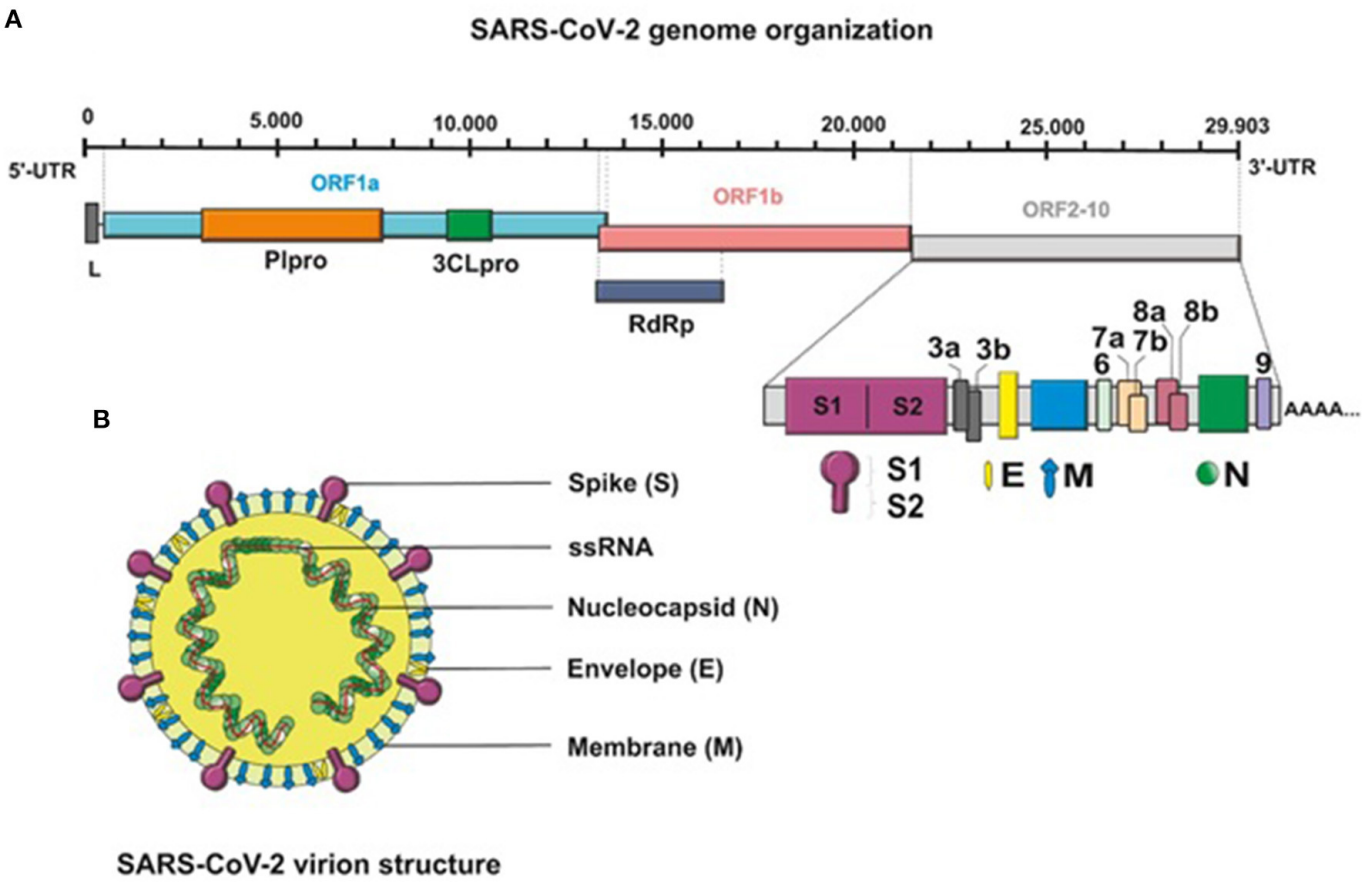
**(A)** Schematic presentation of the full-length genomic RNA (29,903 nt), and transcribed canonical subgenomic RNAs (sgRNA) of SARS-CoV-2 virus. In addition to the genomic RNA that also serves as an mRNA encoding two overlapping ORFs, ORF1a and ORF1b, nine major subgenomic RNAs are produced. **(B)** Schematic structure of the SARS-CoV-2 virion and its major structural proteins.

SARS-CoV-2 is an enveloped, non-segmented, positive-sense single-stranded RNA (+ssRNA) virus that belongs to the genus *Coronavirus*, the family Coronaviridae, and the order Nidovirales (12, 13). Genome sequencing has indicated that the virus shows ~80% similarity to severe acute respiratory syndrome (SARS)-like coronaviruses (SARS-like CoVs) and 50% similarity with Middle East respiratory syndrome coronavirus (MERS-CoV) (14, 15). Structurally, the genome of SARS-CoV-2, with full length of 29,903 bp, is composed of four major structural protein-coding genes namely, spike (S) glycoprotein, envelope (E) glycoprotein, membrane (M) glycoprotein, and nucleocapsid (N) proteins (16). Non-structural proteins are encoded by the open reading frame (ORF) region that consist of replicase complex, which includes components such as 3-chymotrypsin-like protease, papain-like protease, and RNA-dependent RNA polymerase (17) (Figure 1).

## COVID-19 Pathogenesis

**Figure 2:**
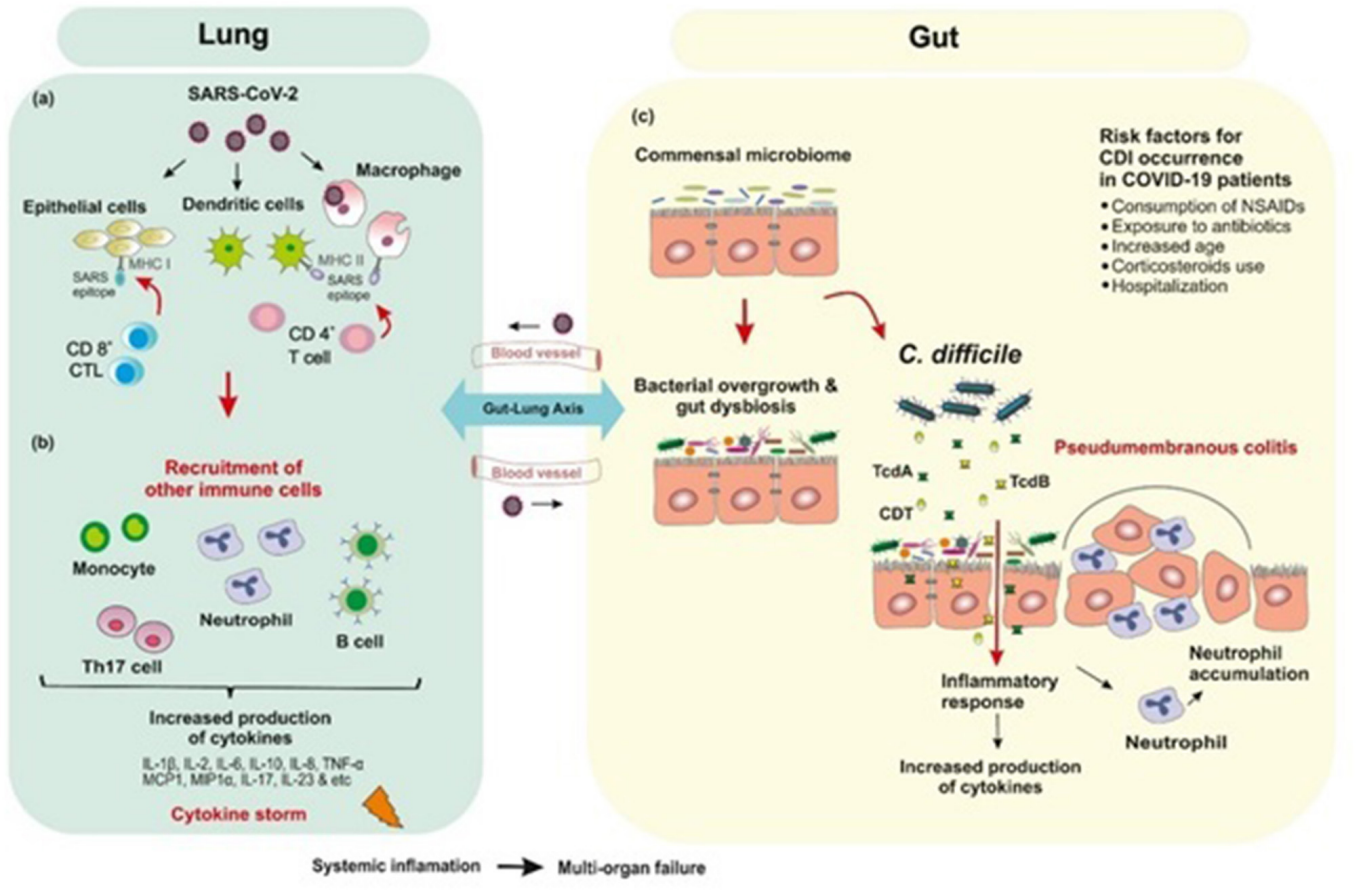
Postulated schematic diagram of the interplay of human gut microbiota and SARS-CoV-2 with bacterial coinfections. **(a)** SARS-CoV-2 binds to ACE2 receptor which mediates direct binding of virus to the human lungs and evokes an immune response. **(b)** Inflammation occurs through the immune responses of both innate and adaptive immune system. CD8^+^ cytotoxic T cells which are able to secret a cluster of molecules are essential in the eradication of virus infected cells. CD4^+^ helper T cells facilitate the overall adaptive response by assisting cytotoxic T cells. B-cell mediated humoral immune response plays a protective role by producing neutralizing antibodies, and also impedes re-infection. SARS-CoV-2 infection triggers the release of inflammatory cytokines and chemokines, which lead to the recruitment of neutrophils and other immune cells. **(c)** Empirical antibiotic therapy for bacterial infections in hospitalized COVID-19 patients are associated with depletion of beneficial commensals and enrichment of opportunistic pathogens such as *Clostridioides difficile* infection. The immune response to *C. difficile* is similar to that produced against SARS-CoV-2 in which inflammatory cytokines are upregulated.

Extensive research around the world has led to a better understanding of the pathogenesis of COVID-19, and development of potential treatments and vaccines against it. Several studies have established that SARS-CoV-2 requires the angiotensin converting enzyme 2 (ACE2) as a major entry receptor (14, 15, 18–20). ACE2 protein, a type I integral metallocarboxypeptidase, is mainly expressed in epithelial and endothelial cells in renal, kidney, lung parenchyma and the GI tract (21). It was shown that glycosylated S proteins, as the most significant transmembrane protein in the outer portion of the virus, bind to the host cell receptor ACE2 and mediate direct binding of virus to the host cells (22, 23). This initial step is an important part of the pathogenesis of COVID-19 infection. Besides, the cleavage of the S proteins by transmembrane protease serine type 2 (TMPRSS2) to subunit S1 and S2 regulates viral uptake and allows the cellular membranes fusion of virus and host cell (24–27). Thus, co-expression of ACE2 and TMPRSS2 is thought to be essential for viral entry into the host cells. Binding to the cellular receptor and inducing membrane fusion lead to the release of uncoated viral RNA genome into the host cell followed by transcription of the sub-genomic RNAs and subsequently, initiation of viral genome replication cycle (12, 28, 29) (Figure 2).

## Immune Response to SARS-CoV-2 Infection

Evidence from genetic analyses indicates that inflammation plays a key role in COVID-19 severity (30). Inflammation occurs through the immune responses of both innate and adaptive immune system; however, it is not clear how this response occurs in SARS-CoV-2 infections. Initially, alveolar epithelial cells, macrophages, and circulating monocytes utilize pattern recognition receptors (PRRs) to detect virus-associated products, this step is followed by recruitment of more immune cells and production of high levels of pro-inflammatory cytokines and chemokines (31). Researchers have hypothesized that the overproduction of early-response pro-inflammatory cytokines such as Interleukin-1β (IL1β), IL-2, IL-6, IL-8, IL-10, tumor necrosis factor-α (TNF-α), interferon γ (IFN-γ), granulocyte colony stimulating factor (G-CSF), monocyte chemoattractant protein-1 (MCP1, also known as CCL2), macrophage inflammatory protein 1 alpha (MIP1α, also known as CCL3), CXC-chemokine ligand 10 (CXCL10), D-dimer, erythrocyte sedimentation rate (ESR), and C-reactive peptide (CRP), in the majority of non-surviving COVID-19 patients contribute to cytokine release syndrome (CRS) or “cytokine storm-induced ARDS”, sepsis, multiple-organ failure and death (20, 30, 32). Among the inflammatory mediators increased in COVID-19 patients, IL-6 blood levels are noticeably highly correlated with mortality (20). Furthermore, there are some reports about the role of T helper 17 (Th17) type cytokine storm in patients with severe COVID-19 (33). Strong Th17 and T-helper 1 (Th1) responses or induced IL-17-related pathways have also been reported in other pulmonary viral infections including MERS-CoV, SARS-CoV, and H1N1 influenza (33, 34) (Figure 2).

## COVID-19 and Gut Microbiota

Alteration of fecal microbiota composition in COVID-19 patients are known to be associated with the enrichment of opportunistic pathogens and depletion of beneficial commensals. Zuo et al. revealed that hospitalized patients with COVID-19 can experience a prolonged dysbiosis in their gut microbiome which was associated with SARS-CoV-2 fecal shedding and disease severity (35). This study showed that several opportunistic pathogens such as *Coprobacillus, Clostridium ramosum*, and *Clostridium hathewayi* were enriched in the gut microbiome of patients with COVID-19. Also, higher abundance of *C. hathewayi* and inverse correlation between abundance of *Faecalibacterium prausnitzii* at the baseline of disease was associated with COVID-19 severity. Nevertheless, over the course of hospitalization, existence of beneficial commensals such as *Bacteroides dorei, Bacteroides thetaiotaomicron, Bacteroides massiliensis*, and *Bacteroides ovatus*, which led to downregulation of ACE2 expression in murine gut, inversely correlated with SARS-CoV-2 load in patients' fecal samples. Thus, it may be speculated that GI tract disease symptoms could be related to the changes in the intestinal microbiome during COVID-19 (35–38). In addition to the bacteria listed above, the prevalence of *Clostridioides difficile* is also dependent on the balance of intestinal microbiota. Recently, it has been shown that disturbing the balance of the gut microbiome called “intestinal dysbiosis” is directly associated with increased risk of *C. difficile* infection (CDI), which in turn, leads to development of intestine-related diseases (39, 40). Furthermore, it has been documented that long-lasting conventional antibiotic administration such as clindamycin, cephalosporins, penicillin, and fluoroquinolones affects the biomass, composition and function of the gut microbiota and consequently reduce colonization resistance capacity against opportunistic pathogens such as *C. difficile*, and subsequently develop CDI (41, 42). Thus, during the current pandemic, clinicians should be vigilant of coinfections in COVID-19 patients with *C. difficile* (Figure 2).

## CDI vs. COVID-19: Comparison of Clinical Manifestations

CDI is an important global infectious disease caused by a toxin-releasing obligate anaerobic organism after long-term use of broad-spectrum antibiotics, absence of antimicrobial stewardship and hospital overcrowding (43–45). Besides, *C. difficile* bacteria produce two major exotoxins, toxin A (TcdA) and toxin B (TcdB), which are both glucosylating toxins responsible for CDI symptoms, including intestinal injury, mucosal inflammation and marked neutrophil recruitment. TcdA and TcdB cause actin cytoskeleton disaggregation, caspases activation, and intestinal cell damage by inactivation of Rho family GTPases (Rho, Rac and Cdc42) (46, 47). CDI spectrum varies from asymptomatic carriage, mild and moderate diarrhea to pseudomembranous colitis, severe fulminant colitis, toxic megacolon, and death, particularly in elderly patients (48, 49).

Annual mortality rate of CDI is high in Europe (8,382 deaths in year) and the United States (29,000 deaths in year) (50). Various risk factors for CDI have been reported such as consumption of different antibiotics, elderly (>65 years), long-term hospitalization, chemotherapy, comorbidities, and chronic kidney and GI disorders (51, 52). It should be noted that these are also factors for COVID-19 which may cause an overlap, and highlights the importance of considering coinfections. The severity of COVID-19 is under the influence of factors such as advanced age (over 65 years) and underlying chronic conditions including chronic respiratory diseases, hypertension, diabetes mellitus, malignancy, and cardiovascular disease (9, 53). Similar GI symptoms including diarrhea, nausea, vomiting and abdominal pain observed in the two diseases would increase the importance of bacterial coinfection in COVID-19 patients.

The cytokines released from immune system during CDI is almost similar to that produced in patients with the severe form of COVID-19. Upregulation of many cytokines mainly IL-1β, IL-6, IL-8, IL-17A, and IL-16, were shown in CDI patients (54). Moreover, the IL-1β/Th17 axis plays a key role in producing inflammatory responses in CDI (54). Notably, elevated Th17 responses affect CDI-associated mortality by increasing IL-17 production. A previous report revealed an association between Th17 cytokines IL-6 and IL-23 and CDI severity (33, 55). These findings demonstrate the key effects of Th17 cells on CDI severity. The role of Th17 cells in patients with severe COVID-19 has also been demonstrated (33).

## Risk Factors Affecting the Occurrence of CDI in COVID-19 Patients

### Possible Risk Factors for *C. difficile* Coinfection

Several studies have confirmed that secondary bacterial infections can increase mortality of the respiratory diseases. During the influenza epidemics, bacterial coinfection was shown to increase the mortality rate of infected-patients (56, 57). Moreover, bacterial infections in intensive care unit (ICU) patients with MERS-CoV were also observed (56). Nonetheless, a limited number of studies have discussed the coinfection of SARS-CoV-2 with bacterial pathogens. This has led to neglect, or underestimation, of the role of bacteria in COVID-19 patients due to the complexity of the symptoms caused by the virus (58).

Some studies have suggested COVID-19 as a possible risk factor for the development of nosocomial infections from *C. difficile* and other multidrug-resistant (MDR) bacteria. Bentivegna et al. (59, 60) showed that during 2020 the use of infection prevention and control (IPC) measures gave rise to a significant decreased of this type of infections in medical departments. Nevertheless, this was not observed in COVID-19 patients where, although not reaching statistical significance, CDI and other nosocomial MDR bacterial infections increased. Since IPC measures were even stricter in the COVID-19 group this apparently conflicting finding opens up several possible interpretations. Some authors, as already mentioned, have suggested the alteration of the intestinal flora in COVID-19 patients as a possible motivation for the increase of CDI (35).

Other possible interpretations suggest the widespread use of broad-spectrum antibiotics in COVID-19 patients as the possible cause of selections and spread of MDR bacteria (61). Impaired immune system, other peculiarities of subjects who develop severe forms of COVID-19 that require hospitalization (62–65), could be another important risk factor for developing CDI. Furthermore, in the hospital unit from which the study data came, COVID-19 patients are mostly managed by infectious disease specialists, who are more likely to perform CD research and culture tests in case of clinical suspicion. Other explanation finds the cause in the intrinsic characteristics of the hospitalized COVID-19 patients itself that are often with pluri-organic comorbidities and several previous hospitalization that could have led to colonization by MDR bacteria. Here, we describe in detail possible risk factors for *C. difficile* coinfection during COVID-19.

### Consumption of NSAIDs

Non-steroidal anti-inflammatory drugs (NSAIDs) such as aspirin, ibuprofen, celecoxib, and indomethacin are widely used for mitigation of pain, inflammation, and fever (48, 66–68). At the early stages of COVID-19, consumption of NSAIDs can help to reduce the fever as the most common symptom of the infected patients. The use of anti-inflammatory drugs and immunomodulators may potentially enhance the prevalence of opportunistic bacteria and predispose development of bacterial coinfections (69, 70). Additionally, it is most likely that elderly (above 65 years old), as an important target population for CDI, are often on multiple medications such as NSAIDs (71). These medications exert their pharmacological effects through reducing the production of prostaglandins (PGs) via inhibition of cyclooxygenase enzymes (COX-1 and COX-2), and modulation of host inflammatory response (72, 73). There are increasing reports indicating the plausibility of an association between use of NSAIDs and increased risk of severe manifestations in CDI (74). Muñoz-Miralles et al. reported that indomethacin-administered, antibiotic-treated mice infected with *C. difficile* demonstrated enhanced intestinal inflammation, weight loss, and presented the signs of severe infection and worsened histopathological damage (75). They concluded that NSAIDs can have a negative impact on antibiotic-associated CDI, and proposed that targeting the biosynthesis or signaling pathways of prostaglandins might be a promising approach to ameliorate the severity of CDI. Moreover, similar results were obtained by Mesada et al. regarding the deteriorate effects of indomethacin exposure on alteration of the gut microbiota and exacerbation of *C. difficile* colitis (76). It was also hypothesized by Noori et al. that the NSAID indomethacin is able to induce the expression of prostaglandin E2-inactivating enzyme named 15-hydroxyprostaglandin dehydrogenase (*Hpgd*, 15-PGDH), which may result in enhanced PGE2 production (77). Furthermore, the therapeutic efficacy, disposition (absorption, distribution, metabolism, and excretion), toxicity and enteropathy of NSAIDs are influenced by the dynamic crosstalk between host cells and gut microbiota (69, 78, 79). On the other hand, NSAIDs can directly alter the structure and functional profiles of the gut microbiota or indirectly impact the physiological status of the host which may, in turn, cause intestinal dysbiosis (80).

Besides, a possible association was reported between NSAIDs, especially diclofenac, and diarrhea among patients with *Clostridium difficile* associated disease (CDAD) that were not hospitalized or exposed to antimicrobial agents (81–83). Suissa et al. found consumption of diclofenac is related with 35% increase in the risk of developing CDAD (81).

### Antibiotic Therapy

The rapid spread of SARS-CoV-2 increased the need to develop effective antiviral agents against COVID-19. Given that there are no clinically approved drugs available for SARS-CoV-2 infection, clinicians have to consider various agents such as antiviral peptides (remidesivir, ribavirin, and favipiravir), antibiotics (azithromycin, doxycycline, clindamycin, cephalosporins, and fluoroquinolone), and different treatment strategies like convalescent plasma transfusion therapy, heparin therapy for improving hypoxia, and also corticosteroids to modulate the inflammation response (84–86). Patients with COVID-19 mostly present with clinical manifestations including fever, cough and lung infiltrates, which resemble bacterial pneumonia. Since cough, fever and radiological infiltrates are common symptoms of pneumonia, appropriate antimicrobial therapy may be required during the hospital stay (87, 88). However, the rate of bacterial coinfection at admission in patients with COVID-19 is extremely low, thus not justifying the extensive use of antibiotics. Therefore, antibiotics should be given only if there is a suspicion of bacterial pneumonia, not because of fever and radiological infiltrates during COVID-19. One of the main causes of bacterial coinfection is the disturbance of microbial balance of the GI tract due to antibiotic exposure and the growth of opportunistic pathogens, such as *Staphylococcus aureus, Streptococcus pyogenes*, and *Pseudomonas aeruginosa* (89).

Generally, maintaining the microbial balance of the GI tract plays an important role in the health of each individual. Furthermore, the indiscriminate use of antibiotics changes the overall diversity and composition of the gut microbiota and disrupts the microbial balance. Disruption of the gut microbiota caused by antibiotics is a risk factor for CDI; however, the entry of *C. difficile* is due to poor infection control measures in the healthcare setting. This is the cause of outbreaks (90, 91). Given that the antibiotic use is a triggering factor associated with the development of CDI, it might be one of the probable consequences of antimicrobial agent's consumption against COVID-19. Also, symptoms of diarrhea caused by *C. difficile* were reported in some patients with COVID-19 (92). This study reported 9 cases of coinfection with COVID-19 and CDI in elderly hospitalized patients who had received antibiotics. In addition, the results of this work showed that the rate of CDI increased from 3.32/10,000 to 3.60/10,000 patient-days during January–April 2020. Examination of patients with *C. difficile* and COVID-19 coinfection showed that both can cause GI symptoms, thus, it is recommended to screen diarrheal patients for CDI during the coronavirus disease pandemic (1). Furthermore, it has been reported that CDI complicates COVID-19 severity, mainly in patients with co-morbidities and previous healthcare exposures (87). These findings once again highlight the significance of infection prevention and control measures, and also antimicrobial stewardship programmers, especially in the management of COVID-19 crisis.

### Older Age

Current epidemiological studies denote comorbidities are significantly associated to increased virus susceptibility and burden of COVID-19 disease (93). As comorbidities often increase with aging, elderly patients are more susceptible to COVID-19 (median age at death 75 years) and may in turn experience a more severe COVID-19 (94). It is also noteworthy that elderly patients over 65 years of age represent approximately 80% of hospitalizations and higher death rates (23-fold) than younger people (95–97). According to the current epidemiological data, the risk of death among SARS-CoV-2-infected patients with comorbidities over 80 years old was greater than younger-aged groups (20, 87, 98–100). A study in China reported that the COVID-19 mortality rate in older patients (>60 years) was significantly higher than that of the younger patients (5.3 vs. 1.4%) (86). A similar study in Italy, as a country most severely affected by COVID-19 pandemic, confirmed the effect of age on the mortality rate of COVID-19 (1). Age-related structural, physiological, and immunological changes of the respiratory system showed that aging itself has been strongly associated with worse health outcomes in COVID-19 patients (94). Moreover, COVID-19 pandemic demonstrated that older people (over 60 years) are not only at a higher risk for severe disease, but they may also be at risk of ignoring their antecedent chronic conditions or being lost to follow up (101). Previous studies suggested that possible decrease in ACE2 expression induced by the SARS-CoV-2 infection in the majority of elderly patients especially those with hypertension may up-regulate angiotensin II, and exacerbate pro-inflammatory conditions, leading to greater COVID-19 severity and mortality (102, 103). Given the disproportionate impact of COVID-19 on older people, age is clearly the most important factor for death due to COVID-19 (104–106). Antibiotic exposure and advanced age (age >65 years old) are two established risk factors for developing CDI (107, 108). It is well known that CDI, is the most important cause of acute diarrhea in all age groups, but it disproportionately affects elderly resulting in increased risk of morbidity and mortality among this compromised population (107, 109). The predisposing risk factors which may be specific to older adults are multifactorial, and include frequent contacts with healthcare systems and age-associated alterations in host physiology such as waning immunity (immunosenescence) and probably altered intestinal microbiota (110, 111).

## Discussion

Despite well-defined recommendation from the WHO for the use of broad-spectrum empirical antibiotics in hospitalized patients with COVID-19 who suffer from bacterial co/superinfections, there had been unjustifiable use of antimicrobials in these patients, especially among those with asymptomatic or mild-to-moderate illness (112). Since pulmonary bacterial super-infections seems to be mostly nosocomial and were associated with receipt of empirical antimicrobial therapy (113), disequilibrium in the composition of human gut microbiota following inappropriate antibiotic consumption might play a prominent role in the emergence of several opportunistic pathogens specially *C. difficile* in longitudinal and high-quality respiratory samples. This is a matter of worrisome when the majority of asymptomatic or hospitalized symptomatic patients with COVID-19 (nearly 72%) were treated with broad-spectrum antibiotics mostly quinolones to prevent bacterial co/superinfections (20, 57, 114). Also, approximately 75% of long-term care facility (LTCF) residents for 6 months or longer will receive at least one course of antibiotics (115). Indeed, alteration of the intestinal microbiome of COVID-19 patients who are treated with broad-spectrum antibiotics, is reported to be associated with escalated clinical status such as proinflammatory conditions, disease severity, fecal shedding of the virus, plasma concentrations of several cytokines and finally drastic long-term effects on host health (35, 116, 117). These traceable alterations of the microbiome of COVID-19 patients following the use of antibiotics can lead to dysbiosis of the microbiome and CDI (118). The use of antibiotics in COVID-19 pneumonia increases the risk of antibiotic-associated diarrhea (AAD) and CDI. Sandhu et al. recently reported a case series of nine patients with coinfection of SARS-CoV-2 and *C. difficile* in hospitalized elderly female patients which were treated with antibiotics. This study emphasizes the unintended consequences of judicious administration of antibiotics throughout the disease course of COVID-19 and bacterial infection such as CDI (92). Additionally, COVID-19 is an illness with frequent GI symptoms such as nausea, vomiting and diarrhea that induces changes in the gut microbiota composition of infected patients, which can complicate the diagnosis of CDI owing to lack of clinical suspicion (119). Not only these patients are more susceptible to CDI, but also antibiotics may induce emergence of new virulent strains of *C. difficile*, which in turn will produce severe consequences in such patients (1). The onset of GI symptoms in COVID-19 patients can be related to the presence of ACE2 receptor and TMPRSS2 in the esophageal upper epithelial, ileum and colon (1), which as previously mentioned, are involved in cell entry of SARS-CoV-2 and its pathogenesis. Studies have shown that ACE2 is the target of SARS-CoV-2 (27, 120) which is highly expressed in colonocytes of patients with inflammatory bowel disease and can regulate amino acid transport, microbial ecology, and inflammation in the gut and create conditions favorable for coronaviruses infection (11, 120). It seems that for prevention of delayed diagnosis of CDI, both CDI and COVID-19 should be considered in patients with GI symptoms (92). Even if GI symptoms are not seen, the possibility of CDI should be considered because of the extensive use of antibiotics. In addition, damage to the gut caused by COVID-19 may facilitate bacterial infection, especially in patients who are susceptible to CDI. Interestingly, some reports showed that beneficial bacterial species are negatively correlated with COVID-19 severity. It has been shown that in bacterial and viral co-infection, commensal species which are able to downregulate ACE2 expression may become potential targets for alleviating immune alterations leading to less severe cases of COVID-19 (121).

Previous data presented that gut microbiome dysbiosis in high-risk groups like those with diabetes, kidney, obesity, autoimmune, and aging-related diseases is often an underlying condition and that GI symptoms are often indicative of severe COVID-19 complication (122). Interestingly, hypovitaminosis D was linked to adverse events in patients with lung, heart, kidney diseases, CDI, and SARS-CoV-2 infection (123–125). Immunological findings confirmed vitamin D involvement in the entire cascade of host responses toward infectious diseases (124, 125). In this regard previous studies have indicated that vitamin D inhibits TNF-α, IFN-γ, IL-1, IL-6, and IL-17, by reducing the p38 MAP kinase activation in human monocytes/macrophages and enhancing the expression level of T-regulatory cells, Th2, M2 macrophages, and IL-10 (126–131). Notably, *C. difficile* stimulate production of inflammatory cytokines such as IL-8, IL-6, IL-1β, TNF-α, COX-2, and PE2, which are suggested playing crucial roles in CDI pathogenesis as well as SARS-CoV-2 infection (132–134). Given that, vitamin D can help prevent upper respiratory tract infections or reduce the severity of them through increasing the immune modulatory activity by inhibition of transcription level of proinflammatory cytokines such as TNF-α, IFN-γ, IL-17, IL-2, and IL-21 (125, 135, 136).

The fecal-oral transmission is one way of shedding *C. difficile* into the environment, which has been confirmed in previous studies (137, 138). Noteworthy, the presence of coronaviruses in GI tract of infected humans has been also proven, so that there are some studies about isolation of live SARS-CoV-2 virus from fecal sample of patients with SARS-CoV-2 respiratory tract infection (139). CDI patients possibly may have facilitated the persistence of SARS-CoV-2 in feces almost a month, even after the nasopharyngeal test turned negative. This coinfection might exacerbate the GI signs and symptoms and raise possibility of fecal-oral transmission of SARS-CoV-2 and *C. difficile* (119, 140). Since SARS-CoV-2 virus can be potentially transmitted through fecal matters even in asymptomatic patients, CDI may complicate COVID-19 and lead to higher rate of mortality (119). Thus, it is needed to clarify the interactions between COVID-19 and CDI. Notably, adherence to health principles including health education and publicity, environmental health and personal hygiene, frequent hand washing, disinfection of surfaces, might become more important in COVID-19 pandemic to prevent occurrence of co-infection (87) and this concern has been addressed by some studies in which, it was emphasized that when CDI is present as a co-infection with COVID-19 monitoring of CDI therapy can be difficult if diarrhea persists because of COVID-19 (59). In addition to the aforementioned points, it is important to note that patients taking proton pump inhibitors (PPIs) are exposed to severe risk of clinical outcomes of COVID-19 (98). Furthermore, there is a statistically significant association between the use of gastric acid suppressors, including PPIs and histamine-2 receptor antagonists (H2RAs) and CDI (47, 99, 100). Since scarce data are available in this context, paying attention to patients receiving PPIs during COVID-19 pandemic may help prevention of CDI coinfection. All of these pieces of evidence highlight the importance of paying attention to CDI during the COVID-19 pandemic. Also, there must be high suspicion for CDI in COVID-19 patients with persistent diarrhea.

## Conclusion

The evidence is available on COVID-19 and CDI coinfection; CDI should be monitored in COVID-19 patients for a better management of the disease. In this regard, we highlight the following risk factors for CDI in COVID-19 patients: (1) The detrimental effect on the gut microbiota caused by antibody therapy in COVID-19 patients should be monitored. (2) Patients with COVID-19 infection who take antibiotics for a long time in the hospital settings or at home may be susceptible to develop CDI. (3) Patients with acute COVID-19 infections who have to be hospitalized for a long time should be monitored for CDI. (4) Adherence to good healthcare practices in medical centers should be considered for COVID-19 patients presenting diarrhea. (5) The importance of CDI diagnosis in patients with GI symptoms to prevent the misdiagnosis of COVID-19 infection. (6) CDI should be considered in COVID-19 patients treated with anti-inflammatory drugs.

Taken together, this review underlines the importance of infection prevention and rational use of antibiotics in the management of patients with COVID-19. Antibiotic treatment can be one of the main causes of this problem, thus, surveillance on antibiotic administration should be incorporated in COVID-19 management.

## Author Contributions

MA, MN, HR, and AY prepared the draft of the manuscript. MN designed the figures. AY, EB, PM, NP, SS, HAA, and MRZ reviewed and revised the manuscript. All authors read and approved the final version of the manuscript and the author list.

## Funding

This work was supported by Foodborne and Waterborne Diseases Research Center, Research Institute for Gastroenterology and Liver Diseases, Shahid Beheshti University of Medical Sciences, Tehran, Iran.

## Conflict of Interest

The authors declare that the research was conducted in the absence of any commercial or financial relationships that could be construed as a potential conflict of interest.

## Publisher's Note

All claims expressed in this article are solely those of the authors and do not necessarily represent those of their affiliated organizations, or those of the publisher, the editors and the reviewers. Any product that may be evaluated in this article, or claim that may be made by its manufacturer, is not guaranteed or endorsed by the publisher.
